# How AI Could Help Us in the Epidemiology and Diagnosis of Acute Respiratory Infections?

**DOI:** 10.3390/pathogens13110940

**Published:** 2024-10-29

**Authors:** Francisco Epelde

**Affiliations:** Internal Medicine Department, Hospital Universitari Parc Taulí, 08208 Sabadell, Spain; fepelde@gmail.com

**Keywords:** artificial intelligence, acute respiratory infections, diagnosis, epidemiology, machine learning

## Abstract

Acute respiratory infections (ARIs) represent a significant global health burden, contributing to high morbidity and mortality rates, particularly in vulnerable populations. Traditional methods for diagnosing and tracking ARIs often face limitations in terms of speed, accuracy, and scalability. The advent of artificial intelligence (AI) has the potential to revolutionize these processes by enhancing early detection, precise diagnosis, and effective epidemiological tracking. This review explores the integration of AI in the epidemiology and diagnosis of ARIs, highlighting its capabilities, current applications, and future prospects. By examining recent advancements and existing studies, this paper provides a comprehensive understanding of how AI can improve ARI management, offering insights into its practical applications and the challenges that must be addressed to realize its full potential.

## 1. Introduction

Acute respiratory infections (ARIs) represent a major global health challenge and are among the leading causes of morbidity and mortality worldwide, posing a severe threat to public health. These infections encompass a broad spectrum of diseases, ranging from the common cold to more severe conditions like pneumonia, influenza, and COVID-19. According to the World Health Organization (WHO), ARIs account for millions of hospitalizations and deaths each year, particularly affecting vulnerable populations such as young children, the elderly, and individuals with pre-existing health conditions.

Timely and accurate diagnosis is imperative, as it significantly influences treatment decisions, the allocation of healthcare resources, and overall patient outcomes. Early identification of the causative agent of an infection can facilitate targeted therapies, reducing the risk of complications and the spread of contagious diseases. Furthermore, effective epidemiological surveillance plays a critical role in understanding and controlling the spread of these infections. Surveillance data is essential for public health authorities to implement timely interventions and allocate resources effectively, ultimately improving health outcomes on a population level.

However, traditional diagnostic methods, such as clinical examinations and laboratory tests, while valuable, often require significant time and resources to yield results. These methods can be limited by factors such as the availability of skilled personnel, laboratory infrastructure, and the turnaround time for test results. The limitations of these conventional approaches are particularly evident during respiratory infection outbreaks, where rapid identification of pathogens is essential for implementing control measures and protecting public health. Additionally, as respiratory infections can have overlapping symptoms, differentiating between various pathogens becomes increasingly challenging without rapid and accurate diagnostic tools.

Moreover, epidemiological tracking heavily relies on manual data collection and reporting, which can be slow, labor-intensive, and prone to human error. Inaccurate data can lead to misinformed public health strategies and delayed responses to emerging threats. This situation has underscored the urgent need for innovative solutions that can enhance diagnostic capabilities and improve data accuracy in public health surveillance [1,2,3].

Artificial intelligence (AI) technologies, with their advanced capabilities for rapid data processing, machine learning, and pattern recognition, offer promising solutions to address these challenges. AI can analyze vast amounts of data quickly, recognize complex patterns that may elude human analysts, and generate predictive models that facilitate proactive public health responses.

In recent years, there has been a growing interest in the application of AI in healthcare, particularly in the diagnosis and management of infectious diseases. Various studies have demonstrated the potential of AI algorithms to improve the accuracy of diagnostic processes, reduce the time required for testing, and enhance the efficiency of epidemiological monitoring. AI has been successfully utilized in areas such as image analysis, biomarker identification, and symptom assessment through natural language processing. By leveraging diverse data sources, including electronic health records, social media trends, and environmental data, AI can provide insights that support early detection and response strategies [4].

This review investigates the potential of AI to enhance the epidemiology and diagnosis of ARIs. By synthesizing findings from various studies, we aim to elucidate the current state of AI applications in this field, identify gaps in knowledge, and propose future research directions. We will explore specific AI methodologies, such as natural language processing, deep learning, and predictive analytics, that can significantly improve diagnostic accuracy and speed. Additionally, we will address the ethical considerations and challenges associated with implementing AI in clinical practice, including issues of data privacy, algorithmic bias, and the importance of integrating AI tools into existing healthcare systems.

Through this examination, we hope to highlight not only the existing advancements but also the challenges that must be overcome to fully realize the potential of AI in transforming the diagnosis and management of acute respiratory infections.

## 2. Methods

This systematic review follows the Preferred Reporting Items for Systematic Reviews and Meta-Analyses (PRISMA) guidelines. A comprehensive literature search was conducted using databases such as PubMed, Google Scholar, and IEEE Xplore, focusing on studies published between 2010 and 2024. Keywords included “artificial intelligence”, “acute respiratory infections”, “diagnosis”, “epidemiology”, and “machine learning”. Studies were selected based on their relevance to the topic, quality of research, and contribution to understanding AI applications in ARIs. A total of 22,913 articles were obtained, and a first review was conducted on 1061 studies considering their methodology, including clinical trials, meta-analyses, and randomized controlled trials [5]. Of the total number of papers obtained, those of greatest relevance were selected for the review presented.

**Figure 1:**
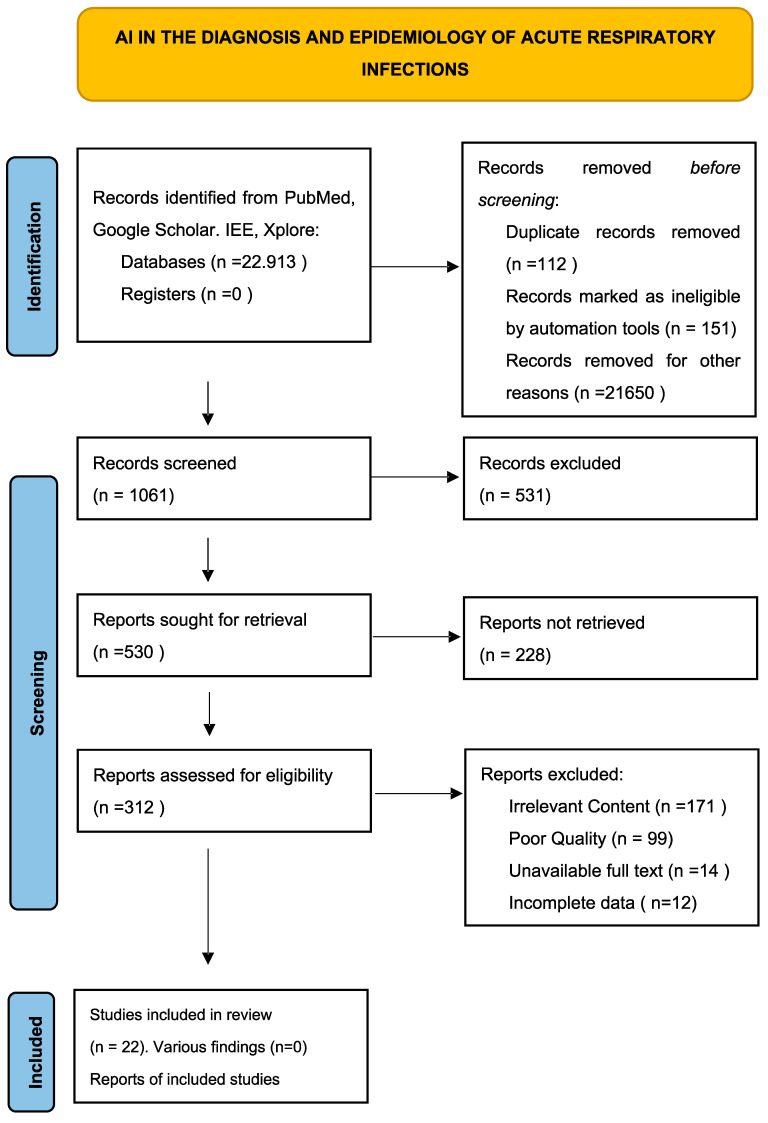
Flowchart of the Study Selection Process for AI Applications in the Diagnosis and Epidemiology of ARIs.

The flow chart of the review of the articles, following the methodology provided by Preferred Reporting Items for Systematic Reviews and Meta-Analyses (PRISMA) (https://www.prisma-statement.org, accessed on 1 October 2024), is shown in Figure 1. as The checklist of items was carried out according to the PRISMA 2020 methodology as can be seen in the file shown in the Supplementary Material shown in Table S1.

## 3. Review Results

### 3.1. AI in the Diagnosis of Acute Respiratory Infections

#### Machine Learning Algorithms

In recent years, machine learning (ML) has emerged as a pivotal tool in the diagnosis and epidemiology of acute respiratory infections (ARIs). Various algorithms have been applied in this field, each offering unique strengths and facing specific limitations that impact their suitability for healthcare applications.

Logistic regression, one of the simplest forms of machine learning, has found its place in clinical practice for binary classification tasks, such as determining the presence or absence of a particular infection based on clinical features. Its interpretability and straightforward approach allow healthcare practitioners to easily understand the probabilities associated with outcomes. However, logistic regression has limitations; it assumes a linear relationship between independent and dependent variables, which may not accurately represent the complexities of ARI data. Additionally, it is sensitive to outliers and can struggle when faced with multicollinearity, where independent variables are highly correlated.

In contrast, decision trees offer a more visual representation of decision-making processes. These algorithms are particularly well-suited for both classification and regression tasks involving categorical data. Decision trees enable clinicians to trace the path of decision-making, which enhances user-friendliness and interpretability. However, they are prone to overfitting, especially when trained on small datasets, and small changes in the data can lead to drastically different tree structures, which may affect reliability.

Random forests, an ensemble method that builds multiple decision trees and aggregates their predictions, enhance the robustness of decision tree models. By combining several trees, random forests typically provide higher accuracy while reducing the risk of overfitting associated with individual trees. This approach is particularly effective in handling large datasets with numerous features, which are common in healthcare. However, the trade-off is a decrease in interpretability; the complexity of the model can make it challenging for healthcare providers to understand the decision-making process.

Support Vector Machines (SVMs) represent another powerful method, especially suited for high-dimensional data. SVMs can effectively classify complex datasets where the classes are not linearly separable by employing various kernel functions. This flexibility often leads to high accuracy in diagnosing ARIs. Nonetheless, SVMs require careful tuning of parameters, such as the choice of kernel and regularization, which can complicate their practical application in clinical settings. Additionally, they can be computationally intensive, particularly when handling large datasets.

Neural networks, particularly deep learning models, have revolutionized the analysis of complex data, such as medical images. Their ability to model intricate nonlinear relationships allows them to excel in feature extraction and classification tasks. While neural networks can achieve high accuracy in diagnosing ARIs, they demand substantial amounts of data and computational resources for training. Moreover, their “black box” nature raises concerns regarding interpretability, which is a significant consideration in clinical environments where understanding the rationale behind a diagnosis is crucial.

Gradient Boosting Machines (GBMs) offer yet another approach, combining multiple weak learners to create a strong predictive model. The GBM has shown excellent performance in various structured data tasks and is particularly effective in improving diagnostic accuracy. However, it is sensitive to overfitting if not carefully optimized, and its complexity can hinder interpretability.

Finally, k-Nearest Neighbors (k-NN) presents a non-parametric approach suitable for classification tasks. This algorithm classifies a sample based on the proximity of neighboring data points, making it straightforward and easy to understand. Despite its simplicity, k-NN can be computationally expensive, especially with large datasets, as it requires distance calculations for each prediction. Additionally, it is sensitive to the choice of distance metric and the parameter k, which can significantly affect its performance.

In summary, the application of machine learning algorithms in diagnosing and tracking acute respiratory infections presents a myriad of opportunities and challenges. While advanced models like deep learning show remarkable potential for accuracy, simpler models such as logistic regression and decision trees retain their importance for interpretability and ease of use in clinical settings. As the healthcare landscape continues to evolve, selecting the appropriate machine learning algorithm will hinge on a careful evaluation of the specific dataset characteristics, the desired outcomes, and the need for transparent decision-making processes.

Machine learning algorithms play a significant role in the diagnosis and epidemiology of respiratory infections by analyzing vast amounts of data to predict outcomes, detect patterns, and provide actionable insights. These algorithms, which include logistic regression, decision trees, random forests, support vector machines (SVMs), neural networks, k-nearest neighbors (k-NNs), and gradient boosting machines (GBMs), have proven effective in various diagnostic tasks [6,7,8].

Logistic regression is widely used in binary classification problems, such as determining whether a patient has a respiratory infection based on symptoms and test results. For instance, it has been employed to predict COVID-19 infection using a combination of symptoms, demographic data, and travel history [9]. Decision trees and random forests are particularly valuable for identifying key factors contributing to the diagnosis and improving prediction accuracy by combining multiple decision trees. These methods have been used in diagnosing pneumonia from patient clinical data, including symptoms, lab results, and medical history [10].

Support vector machines (SVMs) are useful for classifying complex patterns in clinical data, both for binary and multi-class classification problems. For example, they can differentiate between bacterial and viral respiratory infections based on blood test results [11]. Neural networks, especially convolutional neural networks (CNNs), are particularly effective for image-based diagnosis, such as analyzing radiological images. These networks have been used to identify COVID-19 or tuberculosis from chest X-rays or CT scans with high accuracy [12].

K-nearest neighbors (k-NN) is an instance-based learning algorithm that classifies new data points based on the majority class among its k nearest neighbors. This method is particularly useful for predicting the likelihood of a respiratory infection based on similarities to past cases [13]. Gradient boosting machines (GBMs) represent an ensemble technique that builds models sequentially, each correcting errors of the previous ones, thus improving overall accuracy. This approach has been employed to classify types of respiratory infections from comprehensive clinical datasets [14].

Ensemble methods, which combine predictions from multiple machine learning models, have also shown promise in improving diagnostic accuracy and robustness. By integrating different models, ensemble approaches can reduce the variance and bias inherent in individual models, leading to more reliable diagnostic tools. For instance, ensemble methods have been applied to predict the severity of influenza outbreaks by analyzing multiple data sources, including social media trends, clinical records, and environmental factors [15].

Reinforcement learning, a subset of machine learning, is another emerging area with potential applications in ARIs. This technique involves training an AI agent to make decisions by interacting with an environment and receiving feedback in the form of rewards or penalties. Reinforcement learning has been explored for optimizing treatment strategies in patients with ARIs, where the AI agent learns to recommend interventions that maximize patient outcomes [16].

### 3.2. Applications in Real-World Scenarios

Machine learning models have been applied in various real-world scenarios, demonstrating their potential to improve the diagnosis and management of respiratory infections. Early detection is a critical application in which machine learning models analyze initial symptoms and demographic data to predict the likelihood of respiratory infections, enabling early intervention. These models are also used for risk stratification, identifying high-risk patients who may develop severe complications, thereby aiding in targeted healthcare management [17].

Outbreak prediction is another crucial area where time series and spatial models forecast infection trends and hotspots, allowing for timely public health responses. Predictive models play a crucial role in managing acute respiratory infections (ARIs) by enabling healthcare professionals to forecast disease outbreaks, allocate resources effectively, and implement timely interventions. These models utilize historical data, machine learning algorithms, and statistical techniques to analyze trends and make predictions that can inform public health strategies.

One prominent example of predictive modeling is in the realm of vaccination campaigns. By analyzing historical vaccination coverage data alongside infection rates, predictive models can identify populations that are at risk of low vaccination uptake. For instance, health departments can use logistic regression models to assess factors such as demographic information, socioeconomic status, and geographic location, helping to pinpoint communities that may be underserved. This information allows for the design of targeted interventions, such as localized outreach programs and mobile vaccination clinics, ultimately improving vaccine coverage in vulnerable populations.

Another application of predictive models is in forecasting seasonal trends of respiratory infections. Time-series analysis, a statistical technique, is often employed to analyze historical data on infection rates over multiple seasons. For example, by using historical influenza data, health officials can develop models to predict the timing and severity of the upcoming flu season. This predictive capability is critical for preparing healthcare systems, ensuring adequate staffing, and maintaining sufficient medical supplies in anticipation of increased patient volume during peak periods.

Predictive models can also assist in monitoring and controlling outbreaks. Machine learning algorithms, such as random forests and support vector machines, are frequently applied to real-time data, allowing for the rapid identification of potential outbreaks. For instance, during the COVID-19 pandemic, AI models were used to analyze data from various sources, including social media trends, healthcare visits, and geographic information systems, to detect spikes in ARI symptoms. This approach enabled public health officials to implement targeted interventions and allocate resources where they were most needed, thus mitigating the spread of the virus.

Furthermore, predictive modeling has significant implications for individual patient management. In clinical settings, algorithms can be utilized to assess the risk of severe outcomes for patients presenting with respiratory symptoms. For example, machine learning models can analyze patient data, including age, comorbidities, and clinical findings, to estimate the likelihood of hospitalization or the need for intensive care. This information can guide clinical decision-making, ensuring that high-risk patients receive timely and appropriate care.

Lastly, predictive models are essential for assessing the effectiveness of interventions. By employing controlled trials and observational studies, researchers can use statistical models to evaluate the impact of vaccination campaigns or public health measures on infection rates. For instance, cohort studies may implement regression models to assess how vaccination uptake affects the incidence of ARIs in different demographics. Such analyses provide valuable insights into the success of interventions and help refine future strategies.

The application of predictive models in the context of acute respiratory infections is multifaceted, encompassing everything from vaccination strategies to outbreak detection and patient management. By leveraging historical data and advanced analytical techniques, these models empower healthcare professionals to make informed decisions that enhance public health outcomes and improve patient care. These predictive models are instrumental in resource allocation, helping healthcare systems efficiently allocate resources, such as ICU beds, ventilators, and medical staff during surges [18]. Additionally, simulations and predictive analytics inform public health policies, such as social distancing measures, lockdowns, and vaccination strategies, contributing to more effective management of outbreaks [19].

Furthermore, AI has been leveraged in contact tracing efforts during the COVID-19 pandemic. Mobile applications powered by machine learning algorithms have been deployed to identify and notify individuals who have been in close contact with infected persons. These tools have significantly enhanced the speed and accuracy of contact tracing, which is essential for controlling the spread of highly contagious respiratory infections [20].

AI-driven models have also been used in predictive modeling for vaccination campaigns. By analyzing historical data on vaccination coverage and infection rates, AI can help identify populations at risk of low vaccination uptake and design targeted interventions to improve coverage.

These models utilize a variety of machine learning techniques, such as regression analysis, decision trees, and neural networks, to discern patterns and correlations in the data. For example, by examining demographic information, socioeconomic status, and geographic location, AI can pinpoint communities that may be underserved or face barriers to accessing vaccines. This information is crucial for public health officials to effectively allocate resources and tailor their outreach efforts.

Moreover, AI-driven predictive models can simulate various vaccination strategies, evaluating their potential impact on public health outcomes. This capability allows health organizations to test different scenarios, such as varying outreach methods or adjusting vaccination schedules, before implementing strategies in the real world. By forecasting potential outbreaks or the resurgence of vaccine-preventable diseases based on current vaccination rates, AI can facilitate proactive measures to enhance herd immunity within communities.

Additionally, AI can continuously learn from new data as vaccination campaigns progress. This real-time feedback loop enables health authorities to adapt their strategies quickly, responding to emerging trends and challenges. For instance, if a sudden decline in vaccination rates is detected in a specific area, AI systems can trigger alerts, prompting immediate intervention measures to address the issue.

Finally, the integration of AI in vaccination campaigns also supports public awareness initiatives. By analyzing social media trends and public sentiment, AI can inform communication strategies that resonate with target populations, thus enhancing community engagement and participation in vaccination efforts.

AI-driven models not only enhance the efficiency and effectiveness of vaccination campaigns but also contribute significantly to the overall goal of improving public health by ensuring higher vaccination coverage and ultimately reducing the incidence of vaccine preventable diseases.

This application is particularly important in the context of influenza and COVID-19, where high vaccination coverage is crucial for achieving herd immunity [21].

## 4. Challenges and Considerations

While machine learning algorithms offer powerful tools for diagnosing and managing respiratory infections, several challenges must be addressed to fully realize their potential. Data quality and availability are critical issues, as reliable models require high-quality, comprehensive data, which can be challenging to obtain. Furthermore, model interpretability is particularly important in clinical settings where decision-making transparency is crucial [22]. Privacy concerns also arise when handling sensitive health data, necessitating robust measures to ensure patient privacy and data security. Finally, generalizability is a significant concern, as models should be validated across different populations and settings to ensure robustness [23].

The “black box” nature of many AI models, particularly deep learning models, poses a significant challenge to clinical adoption. Clinicians and patients alike may be hesitant to trust AI-driven decisions if they cannot understand the underlying rationale. Therefore, developing explainable AI (XAI) techniques that provide interpretable and transparent models is an ongoing area of research [24].

Another consideration is the potential for algorithmic bias, where AI models may inadvertently learn and perpetuate biases present in the training data. The diagnosis of acute respiratory infections (ARIs) is inherently complex, often leading to variability in diagnostic standards across different healthcare settings. This variability can introduce significant biases that affect patient outcomes and the efficacy of artificial intelligence (AI) systems employed in the diagnosis and management of these infections.

### 4.1. Sources of Bias

One primary source of bias stems from the lack of standardized diagnostic criteria for ARIs. Different healthcare providers may utilize varying clinical guidelines, laboratory tests, and imaging modalities when diagnosing ARIs, leading to inconsistent diagnostic practices. For instance, one facility may rely heavily on rapid antigen tests for influenza, while another may prefer polymerase chain reaction (PCR) testing. Such discrepancies can lead to underdiagnosis in certain populations, particularly in regions where access to advanced diagnostic tools is limited.

Furthermore, the clinical presentation of ARIs can overlap significantly with other respiratory conditions, such as allergies or chronic obstructive pulmonary disease (COPD), leading to misdiagnosis or missed diagnoses altogether. This overlap can result in a reliance on clinical judgment, which is subject to individual biases, potentially exacerbating disparities in care. For instance, patients from marginalized communities may be less likely to receive appropriate diagnoses due to implicit biases held by healthcare providers, leading to higher rates of underdiagnosis in these populations.

The impact of underdiagnosis extends beyond individual patient care; it can affect the data used to train AI models. If certain demographic groups are underrepresented in the data, developed the AI systems may not generalize well to these populations, perpetuating bias and inaccuracies in diagnosis. For example, if an AI model is trained primarily on data from a homogenous population, its ability to accurately diagnose ARIs in diverse patient groups may be compromised, leading to disparities in healthcare delivery.

### 4.2. Ethical and Legal Implications of AI Errors

As AI systems become increasingly integrated into healthcare, understanding the ethical and legal implications of errors made by these systems is crucial. AI algorithms, while powerful, are not infallible. Errors can occur due to biases present in the training data, misinterpretation of patient information, or limitations in the algorithm’s design. Such errors can lead to incorrect diagnoses, delayed treatments, and, in severe cases, preventable morbidity or mortality.

The ethical implications of AI errors primarily revolve around accountability and trust. Patients and healthcare providers must trust that AI systems provide accurate and reliable information to make informed clinical decisions. When errors occur, the question of accountability arises: who is responsible for the harm caused by an AI-driven misdiagnosis? Is it the healthcare provider who relied on the AI system, the developers of the algorithm, or the healthcare institution that implemented the technology? These questions highlight the need for clear accountability frameworks within healthcare organizations.

Establishing accountability frameworks is essential to ensure that stakeholders are held responsible for errors arising from AI systems. These frameworks should outline the roles and responsibilities of healthcare providers, AI developers, and institutions in the event of an error. Moreover, these frameworks must prioritize transparency and patient safety, ensuring that patients are informed about the involvement of AI in their care and understand the potential risks associated with its use.

Additionally, regulatory bodies may need to establish guidelines for the ethical development and deployment of AI technologies in healthcare. This includes defining standards for data quality, bias mitigation strategies, and ongoing monitoring of AI performance. By implementing robust regulatory frameworks, healthcare organizations can promote accountability and trust in AI systems while minimizing the potential for bias and error.

The variability in diagnostic standards and the underdiagnosis of acute respiratory infections can significantly influence biases in AI-driven healthcare systems. Addressing these biases is critical for ensuring equitable healthcare outcomes. Furthermore, establishing clear accountability frameworks is essential to address the ethical and legal implications of errors made by AI systems, fostering a healthcare environment where technology can be harnessed responsibly and effectively.

This issue is particularly concerning in the diagnosis and treatment of ARIs, where biased models could lead to disparities in care across different demographic groups. Ensuring AI models are trained on diverse and representative datasets is crucial for mitigating these risks [25].

Regulatory challenges also need to be addressed for the widespread adoption of AI in healthcare. Regulatory bodies such as the FDA and EMA are still developing frameworks for evaluating and approving AI-based medical devices and algorithms. Ensuring that these frameworks balance innovation with patient safety is essential for fostering trust in AI-driven healthcare solutions [26].

### 4.3. Natural Language Processing

Natural language processing (NLP) techniques enable the extraction of relevant information from unstructured clinical notes and electronic health records (EHRs). By analyzing physicians’ notes and patient histories, NLP algorithms can identify early signs of ARIs and recommend appropriate diagnostic tests. This capability enhances the efficiency and accuracy of diagnosis, particularly in primary care settings where time and resources may be limited [27].

The integration of NLP in healthcare has shown great promise in streamlining the diagnostic process. For example, NLP can be used to automatically extract and categorize symptoms from patient records, which can then be cross-referenced with known indicators of ARIs to provide a preliminary diagnosis. This approach has been successfully implemented in several clinical settings, where it has helped reduce the burden on healthcare professionals and improved patient outcomes [28].

Moreover, NLP has been instrumental in the development of clinical decision support systems (CDSSs). These systems analyze vast amounts of clinical data, including EHRs, medical literature, and treatment guidelines, to provide evidence-based recommendations to clinicians. In the context of ARIs, CDSS powered by NLP can assist healthcare providers in making informed decisions about diagnostic testing, treatment options, and patient management strategies [29].

Another key application of NLP in ARI diagnosis is the automated extraction of epidemiological data from public health reports and scientific literature. By scanning large volumes of text, NLP algorithms can identify emerging trends, track the spread of infections, and provide real-time updates on the status of outbreaks. This capability is particularly valuable during pandemics, where timely access to accurate information is critical for effective public health interventions [30].

### 4.4. AI in Epidemiology of Acute Respiratory Infections

AI has the potential to revolutionize the epidemiological surveillance of ARIs by enabling real-time data collection, analysis, and interpretation. Traditional epidemiological methods often rely on retrospective data analysis, which can delay the identification of emerging trends. In contrast, AI can process large volumes of data from diverse sources, such as electronic health records, social media, and environmental sensors, to provide real-time insights into the spread of respiratory infections [31].

Machine learning models have been used to predict the spread of ARIs by analyzing factors such as population density, weather patterns, and travel data. For instance, AI models have been developed to forecast the incidence of influenza based on historical data and current environmental conditions. These models have shown promise in accurately predicting the timing and severity of flu seasons, enabling public health officials to implement timely interventions [32].

AI has also been used to identify and predict outbreaks of ARIs in real time. By analyzing data from various sources, including social media, news reports, and electronic health records, AI can detect early signs of an outbreak and provide alerts to public health authorities. This capability is particularly valuable for detecting outbreaks in their early stages, when intervention can be most effective in preventing widespread transmission [33].

Furthermore, AI-driven models have been applied to predict the impact of public health interventions on the spread of ARIs. By simulating the effects of different strategies, such as vaccination campaigns, social distancing measures, and travel restrictions, AI can help public health officials make informed decisions about the most effective ways to control the spread of infections [34].

AI’s ability to integrate and analyze data from multiple sources also makes it a powerful tool for tracking the global spread of ARIs. For example, AI models have been used to monitor the spread of COVID-19 across different countries, providing real-time insights into the effectiveness of containment measures and identifying areas at risk of new outbreaks [35]. This global perspective is essential for coordinating international responses to pandemics and ensuring that resources are allocated where they are most needed.

Moreover, AI can assist in identifying risk factors for ARIs by analyzing large datasets that include demographic, behavioral, and environmental variables. By identifying patterns and correlations that may not be immediately apparent to human analysts, AI can help public health officials target interventions to populations at the highest risk of infection [36]. For instance, AI models have been used to identify communities with low vaccination rates and high levels of exposure to environmental pollutants, both of which are risk factors for respiratory infections [37].

Another important application of AI in the epidemiology of ARIs is the development of predictive models for healthcare resource allocation. By forecasting the demand for healthcare services, such as hospital beds, ventilators, and medical staff, AI can help healthcare systems prepare for surges in patient volume during outbreaks [38]. This capability is particularly valuable in resource-constrained settings, where the efficient allocation of limited resources is critical for managing the impact of ARIs [39].

### 4.5. Ethical Considerations

The use of AI in the epidemiology and diagnosis of ARIs raises important ethical considerations. One of the key concerns is the potential for AI models to perpetuate existing health disparities. For example, if AI models are trained on data from populations that are not representative of the broader population, they may produce biased predictions that disadvantage certain groups [40]. Ensuring that AI models are trained on diverse and representative datasets is essential for minimizing the risk of bias and promoting equitable access to healthcare [41].

Another ethical concern is the potential for AI to infringe on patient privacy. The use of AI in healthcare often involves the collection and analysis of large amounts of personal data, which raises concerns about data security and patient confidentiality. It is essential to implement robust data protection measures and ensure that patients are informed about how their data will be used [42]. Additionally, the use of AI in epidemiology may involve collecting data from public sources, such as social media, which raises questions about the ethical implications of using publicly available data for public health surveillance [43].

Furthermore, the use of AI in healthcare decision-making raises questions about accountability and transparency. In cases where AI models are used to make clinical decisions, it is important to ensure that healthcare providers understand how the models work and can explain their recommendations to patients. Additionally, there must be mechanisms in place to hold developers and users of AI systems accountable for their performance and ensure that AI is used in a manner that is consistent with ethical principles [44].

Finally, the use of AI in the epidemiology and diagnosis of ARIs must be guided by the principles of beneficence, non-maleficence, autonomy, and justice. These principles require that AI be used in a way that maximizes the benefits, minimizes harm, respects patient autonomy, and promotes fairness in healthcare access and outcomes [45]. Ensuring that AI is used in a manner that is consistent with these principles is essential for promoting trust in AI-driven healthcare solutions and ensuring that they are used to benefit society as a whole [46].

## 5. Conclusions

This systematic review investigates how AI can enhance the epidemiology and diagnosis of ARIs. In this systematic review, we explored the potential applications of Artificial Intelligence (AI) in the diagnosis and epidemiology of acute respiratory infections (ARIs). AI has shown promising capabilities in improving diagnostic accuracy, reducing the time required for detection, and enabling real-time surveillance of disease outbreaks. Machine learning algorithms, such as neural networks and decision trees, have been successfully applied in diagnosing ARIs through clinical data, imaging, and epidemiological tracking. AI-driven predictive models offer a significant advantage in forecasting infection trends and optimizing resource allocation during outbreaks.

However, several challenges remain. The integration of AI into healthcare systems faces obstacles related to data quality, privacy concerns, and the interpretability of AI models. Additionally, the generalizability of AI tools across different populations and settings is crucial to ensure equitable healthcare outcomes. These challenges must be addressed to fully harness the potential of AI in managing ARIs [47].

Application in Low-Resource Settings: Many areas impacted by ARIs are low-resource settings where access to advanced healthcare technology is limited.

Integration with Public Health Systems: AI can enhance not only individual patient outcomes but also public health responses to infectious disease outbreaks.

By addressing these key areas, AI has the potential to revolutionize the diagnosis, management, and prevention of acute respiratory infections, ultimately improving public health outcomes globally.

## Data Availability

Data will be made available by the authors on request.

## Supplementary Materials

The following supporting information can be downloaded at: https://www.mdpi.com/article/10.3390/pathogens13110940/s1, Table S1: PRISMA 2020 Checklist.
