# Supplementary material for: How AI Could Help Us in the Epidemiology and Diagnosis of Acute Respiratory Infections?
Source: Pathogens. 2024 Oct 29;13(11):940. doi: 10.3390/pathogens13110940 (PMC11597561; doi:10.3390/pathogens13110940)
Supplement: Supplementary file 1 [file pathogens-13-00940-s001.zip › pathogens-3181485-supplementary.pdf]

PRISMA 2020 Checklist

Section and Topic	Item #	Checklist Item	Location where item is reported
**TITLE**			
Title	1	Identify the report as a systematic review.	Title page (Title: "How AI could help us in The Epidemiology and Diagnosis of Acute Respiratory Infections?")
**ABSTRACT**			
Abstract	2	See the PRISMA 2020 for Abstracts checklist.	Abstract (First section in the document)
**INTRODUCTION**			
Rationale	3	Describe the rationale for the review in the context of existing knowledge.	Introduction, paragraph 1 ("Acute respiratory infections...")
Objectives	4	Provide an explicit statement of the objective(s) or question(s) the review addresses.	Introduction, Objectives (Listed in bullet points)
**METHODS**			
Eligibility criteria	5	Specify the inclusion and exclusion criteria for the review and how studies were grouped for the syntheses.	Methods (Section 2)
Information sources	6	Specify all databases, registers, websites, organizations, reference lists, and other sources searched or consulted.	Methods (Search databases: PubMed, Google Scholar, IEEE Xplore)
Search strategy	7	Present the full search strategies for all databases, registers, and websites, including filters and limits used.	Methods (Section 2. Keywords listed)
Selection process	8	Specify the methods used to decide whether a study met the inclusion criteria, how many reviewers screened, etc.	Methods (Section 2, Study selection)
Data collection process	9	Specify methods for collecting data from reports, how many reviewers collected data, and automation tools used.	Methods (Section 2)

Data items	10a	List and define all outcomes for which data were sought.	Results (Section 3)
	10b	List and define all other variables for which data were sought.	Results (Section 3)
Study risk of bias	11	Specify methods used to assess risk of bias in included studies.	Not explicitly mentioned; add section on risk of bias
Effect measures	12	Specify for each outcome the effect measure(s) (e.g., risk ratio, mean difference) used in the synthesis.	Results (Section 3)
Synthesis methods	13a	Describe processes used to decide which studies were eligible for synthesis.	Methods (Section 2, synthesis approach not fully described)
	13b	Describe methods for preparing data for presentation or synthesis (handling missing data).	Methods (Section 2, data synthesis section can be added)
	13c	Describe methods for tabulating or visually displaying results.	Results (Table, Figures, or Chart missing)
	13d	Describe methods used to synthesize results, including meta-analysis.	Results (Section 3)
	13e	Describe methods to explore heterogeneity (e.g., subgroup analysis).	N/A (Not applicable in your review)
	13f	Describe sensitivity analyses conducted.	N/A
Reporting bias assessment	14	Describe methods to assess risk of bias due to missing results (reporting bias).	N/A (No section on bias)
Certainty assessment	15	Describe methods to assess certainty in the evidence for each outcome.	Results (Section 3)
**RESULTS**			
Study selection	16a	Describe the results of the search and selection process; include a flow diagram.	Methods (Flowchart missing)
	16b	Cite studies excluded, explain why excluded.	Not explicitly mentioned; missing section
Study characteristics	17	Cite each included study and present its characteristics.	Results (Section 3)

Risk of bias in studies	18	Present risk of bias assessments for each included study.	N/A (Risk of bias section missing)
Results of individual	19	Present summary statistics for each study and effect estimates.	Results (Section 3)
Results of syntheses	20a	Summarize the characteristics and risk of bias among contributing studies.	N/A
	20b	Present statistical synthesis results, including meta-analysis.	Results (Section 3)
	20c	Present results of heterogeneity investigations.	N/A
	20d	Present sensitivity analyses.	N/A
Reporting biases	21	Present assessments of reporting bias for each synthesis assessed.	N/A
Certainty of evidence	22	Present certainty or confidence assessments for each outcome.	Results (Section 3)
**DISCUSSION**			
Discussion	23a	Provide a general interpretation of the results in the context of other evidence.	Discussion (Section 4)
	23b	Discuss limitations of the evidence included in the review.	Discussion (Section 4)
	23c	Discuss limitations of the review processes used.	Discussion (Section 4)
	23d	Discuss implications of the results for practice, policy, and future research.	Discussion (Section 4)
**OTHER INFORMATION**			
Registration and protocol	24a	Provide registration information for the review.	Not mentioned (consider adding)
	24b	Indicate where the review protocol can be accessed.	Not applicable
	24c	Describe and explain any amendments to registration or protocol information.	N/A

Support	25	Describe sources of financial or non-financial support for the review.	Not mentioned
Competing interests	26	Declare any competing interests of review authors.	Not mentioned (consider adding)
Availability of data, code	27	Report availability of data, code, and materials used in the review.	N/A
